# 

**Published:** 1967-04

**Authors:** 


### BRISTOL MEDICO-CHIRURGICAL JOURNAL

*(Incorporating the Medical Journal of the South-West)*

Established 1883

Vol. 82 (ii) April, 1967 No. 304

### Contents

| | | Page |
|---|---|---|
| CAVE DIVING HAZARDS ........................ | *Oliver C. Lloyd* | 23 |
| PICTORIAL ART IN MEDICINE ........... | *G. M. Fitzgibbon* | 35 |
| HAEMOLYTIC DISEASE OF THE NEWBORN WITH PULMONARY ATELECTASIS: A Clinico-Pathological Conference | | 46 |
| ON THE CHANGING FACE OF RHEUMATISM | *Professor C. B. Perry* | 54 |
| LONG TERM PROGNOSIS IN CLOSED HEAD INJURY | *C. Adey* | 58 |

*PUBLISHED QUARTERLY*

*ANNUAL SUBSCRIPTION* **16/-** *POST FREE*

### BRISTOL MEDICO-CHIRURGICAL SOCIETY

President 1966-67: Dr. S. F. Marwood.

President-elect: Professor T. F. Hewer.

Honorary Secretary: Dr. A. T. M. Roberts, 8 Harley Place, Bristol 8.

Honorary Treasurer: Dr. J. Macrae, Ham Green Hospital, Pill.

The Society was founded in 1874. In 1883 publication of this Journal began and has continued quarterly since then. During the years 1949 to 1962 it appeared under the title of "The Medical Journal of the South West".

The Society founded a medical library in 1890, which in 1892 was combined with that of the Bristol Medical School. This became the University of Bristol Medical Library in 1925, and an agreement in that year confirmed the privilege of Members to use the University Medical Library.

### THE BRISTOL MEDICO-CHIRURGICAL JOURNAL

*Editorial Committee*

Dr. N. J. Brown, Southmead Hospital, Bristol. *Joint Editor.*

Dr. A. B. Raper, Department of Pathology, Bristol Royal Infirmary. *Joint Editor*

Dr. J. Macrae, Ham Green Hospital, Pill, Bristol. *Honorary Treasurer.*

Dr. R. C. Wofinden, Central Clinic, Bristol.

Mr. A. E. S. Roberts, Medical School Library, University Walk, Bristol 8. *Secretary*

### NOTICE TO CONTRIBUTORS

The Journal is especially concerned to provide a place for the recording of medical thought, interests, and practice in and around Bristol. It therefore welcomes articles that, as well as being of immediate interest to members, will document the local and contemporary medical scene for our successors.

Original articles are invited provided that they have not been published elsewhere. References in the text should b by author's name, with year of publication in parenthesis; they should be listed at the end in alphabetical order. Illustrations should be supplied ready for reproduction. Line drawings should be in indian ink on firm white paper or board. The author will be informed if it is found necessary to ask him to pay for the making of blocks. The Editorial Committee reserves the right to make literary corrections.

The following contributions will be especially welcome: notes of forthcoming events, meetings held, degrees conferred, staff appointments, honours and public appointments and other local news.

### LOCAL MEDICAL NEWS

**Bristol Children's Hospital**

Queen Elizabeth the Queen Mother opened the new Out-patients Department, Department of Child Health, Radiology and Pathology Departments on October 27th, 1966.

**Appointments**

Mr. Norman C. Tanner, senior Surgeon at Charing Cross Hospital, has been made an Honorary Fellow of the American College of Surgeons.
Dr. F. G. Farrelly has been appointed Psychiatrist, Ministry of Labour, Health and Housing, Guyana.
Dr. I. D. Fraser has been appointed Consultant Pathologist, South Somerset Clinical Area (Taunton).

**Obituary**

Hubert Chitty, formerly honorary consultant surgeon at Winford Hospital, died on 13th December 1966, aged 84.

### EXAMINATION RESULTS

University of Bristol, Faculty of Medicine

| | |
|---|---|
| M.D. | J. R. Coleman |
| | S. C. Jordan |
| Ph.D. | K. Jayasena |
| | R. H. Johnson |
| | H. P. Khataniar |

College of Pathologists

| | |
|---|---|
| M.C. Path. | Sheila A. Faint |
| | P. M. Keane |

